# Guanfacine inhibits interictal epileptiform events and sodium currents in prefrontal cortex pyramidal neurons

**DOI:** 10.1007/s43440-023-00458-4

**Published:** 2023-02-17

**Authors:** Michał Pasierski, Weronika Kołba, Bartłomiej Szulczyk

**Affiliations:** Department of Pharmacodynamics, The Medical University of Warsaw, Banacha 1B, 02-097 Warsaw, Poland

**Keywords:** Attention deficit hyperactivity disorder, Interictal discharges, Guanfacine, Sodium channels, Patch-clamp

## Abstract

**Background:**

Guanfacine (an alpha-2A receptor agonist) is a commonly used drug with recognized efficacy in the treatment of attention deficit hyperactivity disorder (ADHD). This study aimed to assess the effects of guanfacine on short-lasting (interictal) epileptiform discharges in cortical neurons. Moreover, we assessed the effects of guanfacine on voltage-gated sodium currents.

**Methods:**

We conducted patch-clamp recordings in prefrontal cortex pyramidal neurons obtained from young rats. Interictal epileptiform events were evoked in cortical slices in a zero magnesium proepileptic extracellular solution with an elevated concentration of potassium ions.

**Results:**

Interictal epileptiform discharges were spontaneous depolarisations, which triggered action potentials. Guanfacine (10 and 100 µM) inhibited the frequency of epileptiform discharges. The effect of guanfacine on interictal events persisted in the presence of alpha-2 adrenergic receptor antagonist idazoxan. The tested drug inhibited neuronal excitability. Tonic NMDA currents were not influenced by guanfacine. Recordings from dispersed neurons showed that the tested drug (10 and 100 µM) inhibited persistent and fast inactivating voltage-gated sodium currents.

**Conclusions:**

This study shows that guanfacine inhibits interictal discharges in cortical neurons independently of alpha-2A adrenergic receptors. This effect may be mediated by voltage-gated sodium currents. Inhibition of interictal activity by guanfacine may be of clinical importance because interictal events often occur in patients with ADHD and may contribute to symptoms of this disease.

**Graphical abstract:**

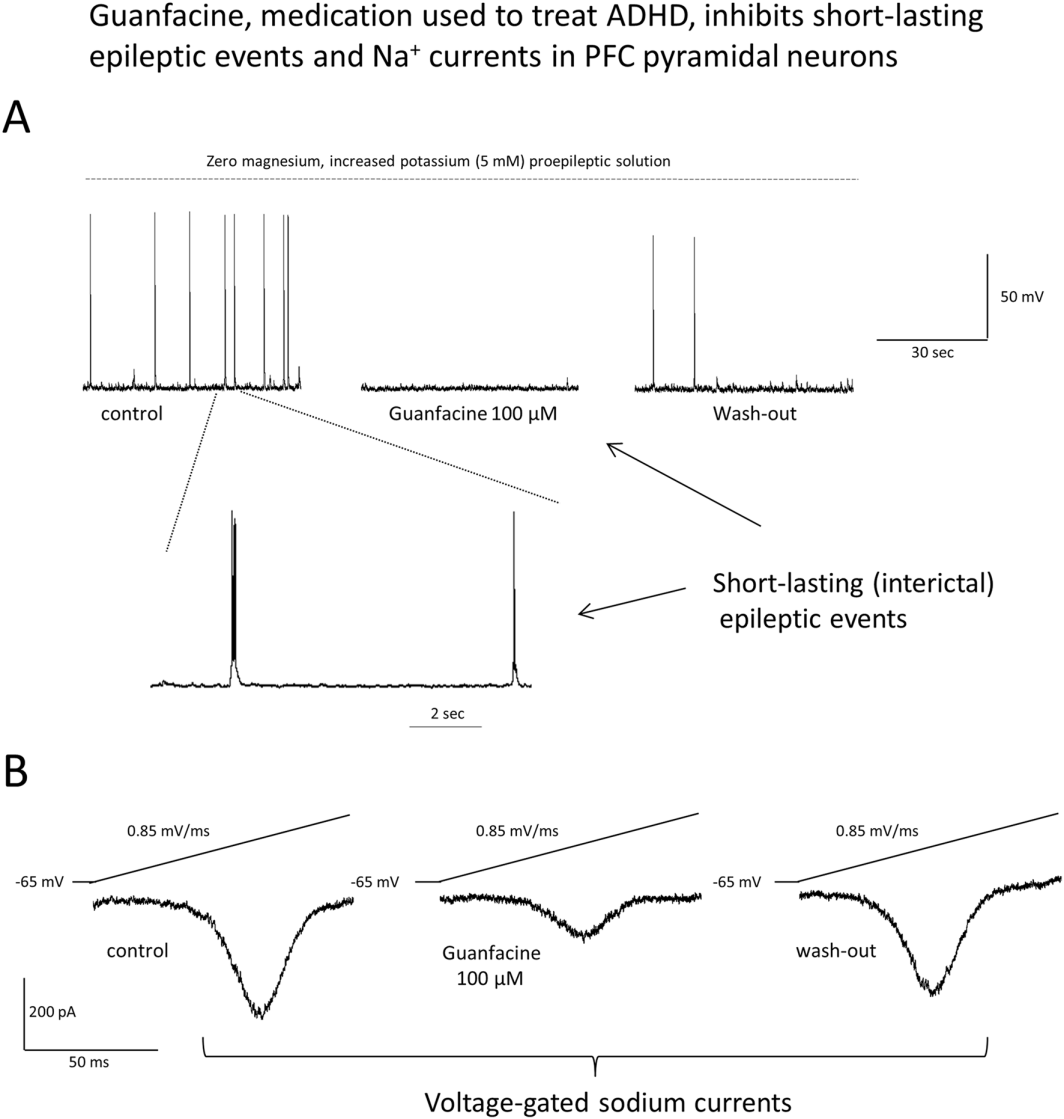

**Supplementary Information:**

The online version contains supplementary material available at 10.1007/s43440-023-00458-4.

## Introduction

Attention deficit hyperactivity disorder (ADHD) is described as a persistent pattern of inattention and/or hyperactivity-impulsivity that interferes with functioning or development [1]. Emerging evidence points to the involvement of the prefrontal cortex (PFC) in the pathogenesis of ADHD [2]. One study showed that children with ADHD present with a slowed or impaired development of the right lateral PFC [3]. Other authors have described a smaller volume of grey matter in the PFC of boys with ADHD as compared to age-matched controls [4]. Consequently, the PFC is a target for ADHD medications [5]

Guanfacine, an alpha-2A-adrenergic receptor agonist, is an approved medication for ADHD, both in adults and children [6]. It has been shown that guanfacine and other ADHD medications such as methylphenidate improve cognitive functions which are impaired in ADHD [5, 6]. In the PFC, guanfacine enhances cognition by stimulating post-synaptic alpha-2A-adrenergic receptors located in pyramidal neurons. This leads to the inhibition of nearby cAMP-dependent K^+^ channels, which strengthens network connectivity and working memory [6]. Besides ADHD, guanfacine has been used “off-label” in several other conditions associated with prefrontal cortex pathology, such as autism spectrum disorders (ASD), schizophrenia, substance abuse, and post-traumatic stress disorder [6].

Several reports have linked epileptic seizures with a decline in cognitive performance, which is also a feature of attention and social disorders [7, 8]. Less attention has been focused on interictal epileptiform discharges (IEDs) or subclinical epileptiform activity which occurs in epileptic patients between seizures [7, 9]. These types of epileptiform events may also be present in patients without seizures [10]. Interictal epileptiform discharges are a relatively common finding in patients diagnosed with ADHD and are presumed to have a causal relationship with the symptoms of the disease [10–15]. Cognitive impairment is a common symptom of ADHD. From a pathophysiological standpoint, increased excitability associated with IEDs may hinder cognition through calcium excitotoxicity, remodeling of neuronal circuitry, or disruption of sleep-related memory consolidation processes [7] There have been reports suggesting that targeting epileptiform activity may alleviate cognitive problems [7, 11, 12].

Our previous publication showed that a common anti-epileptic drug—valproate, a sodium channel blocker and a GABA receptor agonist, inhibits interictal activity in the prefrontal cortex [16]. In the current report, we aimed to assess the effect of guanfacine on interictal activity in the rat’s prefrontal cortex in vitro. Furthermore, we sought to investigate the mechanism of this effect.

## Materials and methods

The experimental procedures used in this study adhered to the Polish and international guidelines on the ethical use of animals (Directive 2010/63/EU, Polish Legislation for the protection of animals used for scientific or educational purposes 2015). Male Wistar Rats (3 week-old) were purchased from the Medical University of Warsaw animal house. The total number of animals used in this study was 20. Rats were bred at room temperature (3 rats per cage, 12 h/12 h light/dark cycle) and fed with a standard laboratory chow. After decapitation, the brain was gently removed. Decapitation was the only procedure performed on animals in this study. Slices (300 µM) of the prefrontal cortex were prepared exactly the same way as shown in our previous publication [16]. After cutting, slices were incubated in a physiological artificial cerebrospinal fluid (ACSF) of the following composition (in mM): NaCl (130), KCl (2.5), glucose (10), NaHCO_3_ (25), NaH_2_PO_4_ (1.25), MgCl_2_ (1), and CaCl_2_ (2), pH = 7.4, bubbled with carbogen. For the experiments in slices this solution was heated to 32 °C for the first 20 min of incubation and after that was maintained at room temperature. For the recordings from dispersed neurons, the incubating solution was maintained at room temperature for the duration of the experiment (6–8 h).

### Recordings in slices

Recordings were made from layer V pyramidal neurons in slices of the medial prefrontal cortex.

Action potentials and IEDs were recorded in the current-clamp configuration. Action potentials were evoked once every 60 s by 250 pA rectangular current steps lasting 3 s in physiological ACSF (see above). Interictal epileptiform discharges (IEDs) were recorded in zero magnesium/5 mM potassium proepileptic extracellular solution which contained (in mM): NaCl (130), KCl (5), glucose (10), NaHCO_3_ (25), NaH_2_PO_4_ (1.25), and CaCl_2_ (2), pH = 7.4, bubbled with carbogen. IEDs were spontaneous discharges and were recorded in membrane potential recording mode.

Tonic NMDA currents were recorded in the voltage-clamp configuration in zero magnesium extracellular solution which contained (in mM): NaCl (130), KCl (2.5), glucose (10), NaHCO_3_ (25), NaH_2_PO_4_ (1.25), and CaCl_2_ (2), glycine (0.05), pH = 7.4, bubbled with carbogen. Magnesium ions were omitted and glycine was added to facilitate NMDA receptors. Moreover, this solution contained, tetrodotoxin (TTX) 0.25 µM, DNQX (6,7-Dinitroquinoxaline-2,3-dione) 10 µM and picrotoxin 50 µM to block synaptic transmission. NMDA 2 µM was applied to the bath. After stable NMDA current was evoked, NMDA 2 µM and guanfacine 100 µM were coapplied (see results).

For all slice recordings, the intracellular solution in the patch pipette was composed of (in mM): potassium-gluconate (105), KCl (20), HEPES-Na + (10), EGTA (0, 1), MgATP (4), GTP (0.5), pH = 7.4. Neurons were visualized in DIC optics. Slice recording techniques were the same as in our previous study [16]. Positive pressure was applied to the pipette tip to blow away extracellular debris. After gigaseal formation, the patch membrane was ruptured. Recordings were made using a Multiclamp 700A amplifier and analyzed with pClamp software (Axon Instruments, USA). Patch-pipettes had resistances between 4 and 5 MΩ. Recordings were obtained at 35 °C. Guanfacine was applied to the bath.

### Recordings in dispersed neurons

Sections of slices containing the prefrontal cortex were enzymatically dispersed using protease type XIV (0.5 mg/ml) and mechanically dispersed using Pasteur pipettes exactly the same way as in our previous publication [17]. Dispersed neurons were transferred to a recording chamber. Recordings were made from pyramidal neurons which were visualized under an inverted microscope.

Persistent voltage-gated sodium currents were recorded in an external solution that contained the following (in mM): NaCl (120), CaCl_2_ (2), MgCl_2_ (2), TEA-Cl (30), 4-AP (3), HEPES (10), glucose (15), CdCl_2_ (0.4), LaCl_3_ (0.005), pH 7.4. Fast activating and fast inactivating voltage-gated sodium currents were recorded in an external solution of the following composition (in mM): NaCl (30), choline chloride (90), TEA-Cl (30), CaCl_2_ (2), MgCl_2_ (2), glucose (15), HEPES (10), CdCl_2_ (0.4) and LaCl_3_ (0.005), at pH 7.4. Voltage-gated calcium currents were blocked by cadmium and lanthanum ions in the extracellular solution. Voltage-gated potassium currents were blocked by TEA-CL in the extracellular solution. Moreover, potassium ions were absent in the intracellular solution. The pipette (intracellular) solution was the same for fast and persistent sodium currents and contained the following (in mM): CsF (110), NaCl (7), EGTA (3), HEPES-Cl (10), MgCl_2_ (2), Na_2_ATP (4), pH was 7.4.

Recording techniques were exactly the same as in our previous study [17]. After gigaseal formation, the membrane was ruptured. The access resistance ranged from 5 to 7 MΩ. A series resistance compensation of 80% was applied. The currents were leak subtracted. All recordings were performed at room temperature (21–22 °C). Currents were recorded using an Axopatch 1D amplifier and analyzed with pClamp software (Axon Instruments, USA). Guanfacine was applied to the bath.

Guanfacine was purchased from Sigma-Aldrich (product number G1043). DNQX, picrotoxin NMDA and idazoxan were also purchased from Sigma-Aldrich (product numbers D0540, P1675, M3262 and I6138, respectively). Tetrodotoxin (TTX) was purchased from Abcam (product number ab120055). Other chemical compounds were purchased from Polskie Odczynniki Chemiczne Avantor or from Sigma-Aldrich.

### Statistical analysis

Normally distributed values are presented as means ± SEM, whereas non-normally distributed values are as medians [IQR]. Differences between more than two groups were evaluated using one-way ANOVA for repeated measures followed by the Tukey post hoc test if the data passed the normality test. If the data did not pass the normality test nonparametric equivalent of one-way ANOVA for repeated measures (Friedman’s test) followed by Dunn’s post hoc test was used. Depending on the results of the normality test, the Students *t* test or Wilcoxon matched-pairs test were used to evaluate differences between the two groups. Kolmogorov–Smirnov test was used to assess normality (GraphPad InStat software v3.06).

## Results

### Induction of interictal epileptiform events in PFC pyramidal neurons

**Fig. 1:**
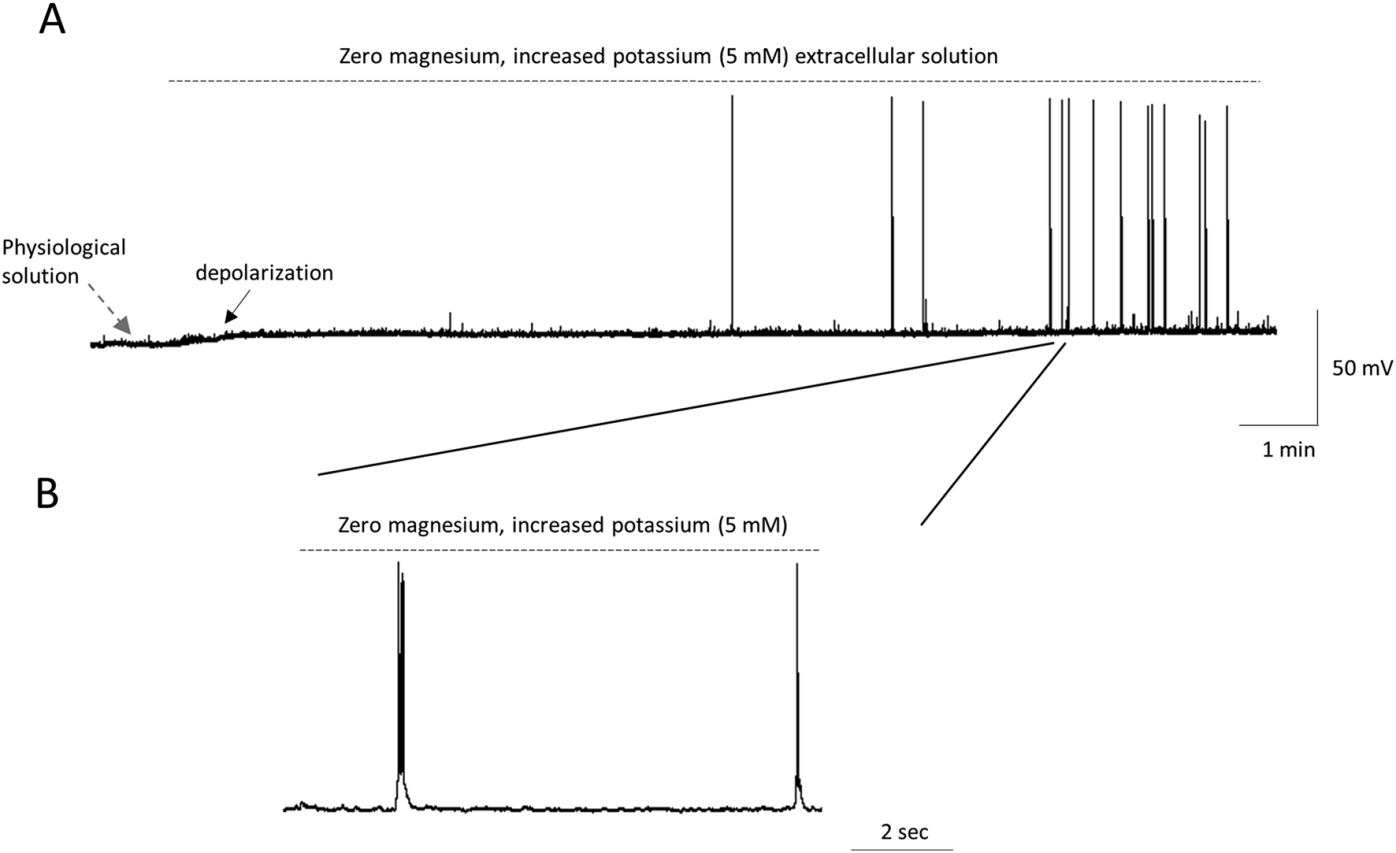
Induction of interictal epileptiform events (IEDs) in prefrontal cortex pyramidal neurons. **A** IEDs were induced in a zero magnesium and high potassium proepileptic extracellular solution as shown by a dashed line. The membrane potential was initially recorded in physiological artificial cerebrospinal fluid, as shown by the grey dashed arrow. Application of the proepileptic extracellular solution depolarized the membrane potential because of an increased potassium ions concentration, as shown by the black solid arrow. **B** Two IEDs are shown on an expanded time scale. The same vertical scale is used for (**A**) and (**B**)

Interictal epileptiform discharges (IEDs) were evoked in a zero magnesium, high potassium proepileptic extracellular solution similar to our previous study [16]. Firstly, the membrane potential was stabilized in a physiological ACSF for a few minutes. After switching to the proepileptic solution, the membrane potential depolarised because of increased potassium ions concentration (− 66.6 ± 2.3 mV and − 59.8 ± 3.2 mV in a physiological and proepileptic solution, respectively, *n* = 6 recordings, the paired Students *t* test, *p* = 0.0362, *t*_5_ = 2.8, as shown by the black solid arrow in Fig. 1A). After 10–30 min of applying the zero magnesium, high potassium proepileptic extracellular solution, IEDs were evoked which were brief depolarisations capped by action potentials (Fig. 1A and B). The events lasted less than 2 s [9, 16]. Two single epileptiform events are shown on an expanded time scale in Fig. 1B.

### Guanfacine inhibits interictal epileptiform events in PFC pyramidal neurons

After a steady control frequency of the IEDs was recorded, the influence of guanfacine on the membrane potential and the frequency of interictal events were assessed. Guanfacine was applied for 7–10 min. The tested compound (100 μM) did not change the membrane potential (− 58.8 ± 2.3 mV in the control and − 59.7 ± 2.7 mV after the application of the drug, *n* = 5 recordings, the paired Students *t* test, *p* > 0.05, *t*_4_ = 1.04).

**Fig. 2:**
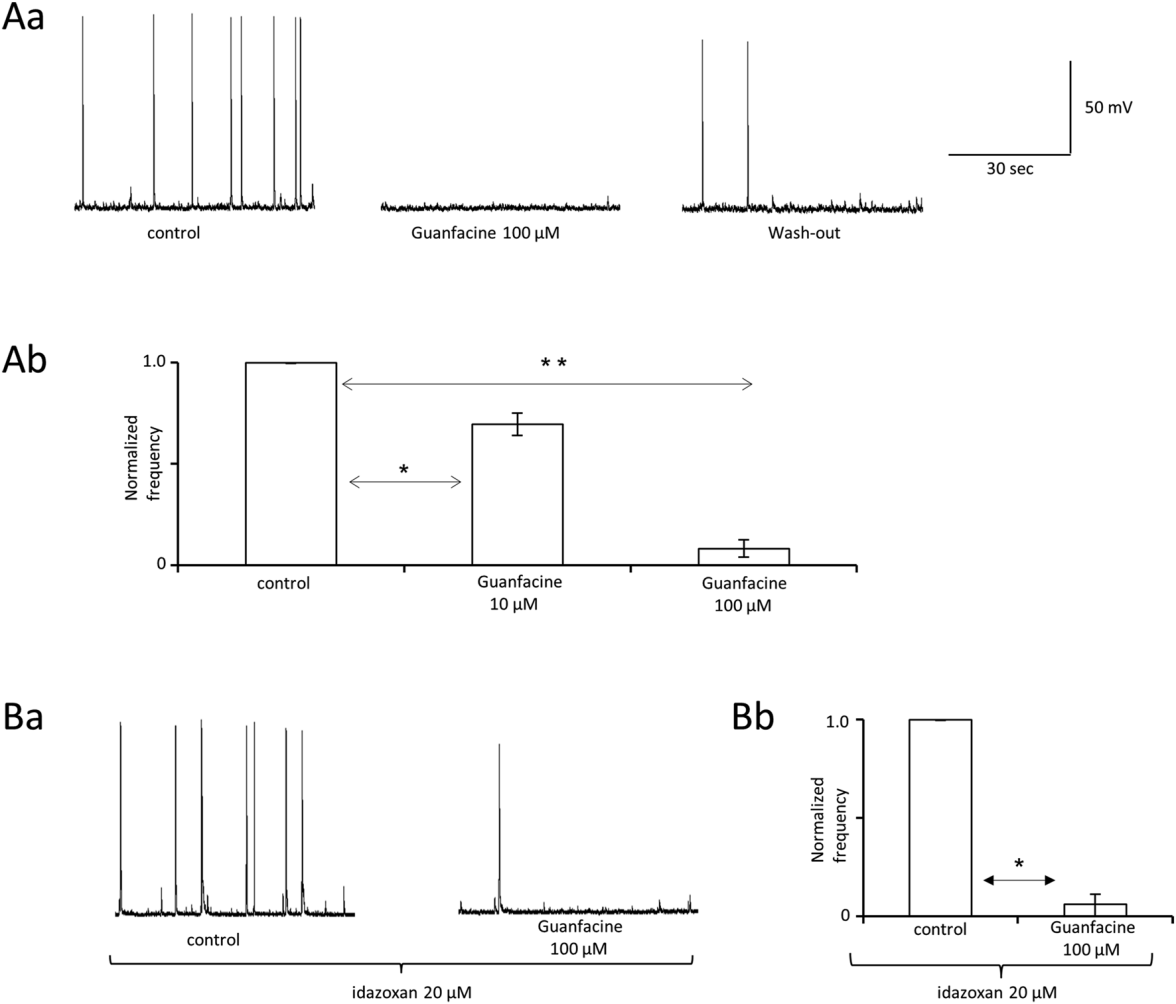
Guanfacine inhibits interictal epileptiform events (IEDs) in prefrontal cortex pyramidal neurons. **Aa** Original recordings of IEDs in control, in the presence of guanfacine 100 µM and after wash-out. **Ab** Normalized frequency of IEDs in the control and in the presence of guanfacine: 10 µM (paired t test [control vs drug application], *p* < 0.05) and 100 µM (one-way ANOVA for repeated measures [control, drug application, wash-out], *p* < 0.0001, followed by Tukey’s post hoc test [control vs drug application], *p* < 0.001). Bars represent means and whiskers represent SEM. *p < 0.05 and **p < 0.001. **Ba** Original recordings of IEDs in the control and after application of guanfacine 100 µM (alpha-2 adrenergic receptor antagonist idazoxan 20 µM was present in all extracellular solutions). **Bb** Normalized frequency of IEDs in the control and in the presence of guanfacine. Idazoxan was present in all extracellular solutions (Friedman’s test [control, drug application, wash-out], *p* = 0.0046, followed by Dunn’s post hoc test [control vs drug application], *p* < 0.05). Inset in (**Aa**) applies to (**Ba**). Bars represent medians and whiskers represent IQR. *p < 0.05

The control frequency of the IEDs was 0.12 ± 0.02 Hz (*n* = 15). Guanfacine inhibited the frequency of the IEDs. Example recordings are shown in Fig. 2Aa. The normalized frequency of the IEDs was 1.0 ± 0.0 in the control, 0.08 ± 0.04 in the presence of guanfacine (100 μM), and 0.33 ± 0.04 after wash-out (3 animals, *n* = 6 recordings, one-way ANOVA for repeated measures, *F*_3,17_ = 324.72 (*p* < 0.0001) followed by Tukey’s post hoc test (*p* < 0.001 control vs guanfacine), Fig. 2Ab). As expected, a lower concentration of guanfacine (10 μM) inhibited the normalized frequency of the IEDs to a smaller extent (1.0 ± 0.0 in the control compared to 0.69 ± 0.05 after the application of the drug, *n* = 5 recordings, 2 animals, the paired Students *t* test, *p* = 0.0042, *t*_4_ = 5.9, Fig. 2Ab).

The most commonly described mechanism of action of guanfacine is the stimulation of alpha-2A adrenergic receptors [18]. For this reason, we assessed the influence of guanfacine 100 μM on the frequency of IEDs in the constant presence of the alpha-2 adrenergic receptor antagonist idazoxan in all extracellular solutions. With idazoxan (20 μM) in the bath, guanfacine 100 μM inhibited the frequency of the IEDs to the same extent as without idazoxan in the bath (see above). Example recordings are shown in Fig. 2Ba and normalized results are shown as medians in Fig. 2Bb 1.0 [1.0–1.0] in the control, 0.06 [0.00–0.11] after the application of guanfacine 100 μM and 0.36 [0.26–0.52]) after wash-out, 2 animals, *n* = 4 recordings, nonparametric repeated measures ANOVA (Friedman’s test), Friedman’s statistic = 8, *p* = 0.0046 followed by Dunn’s post hoc test (*p* < 0.05, control vs guanfacine)). Thus, the effect of guanfacine on IEDs in PFC pyramidal neurons is independent of alpha-2A adrenergic receptors.

### Guanfacine inhibits neuronal excitability and voltage-gated sodium currents in PFC pyramidal neurons

**Fig. 3:**
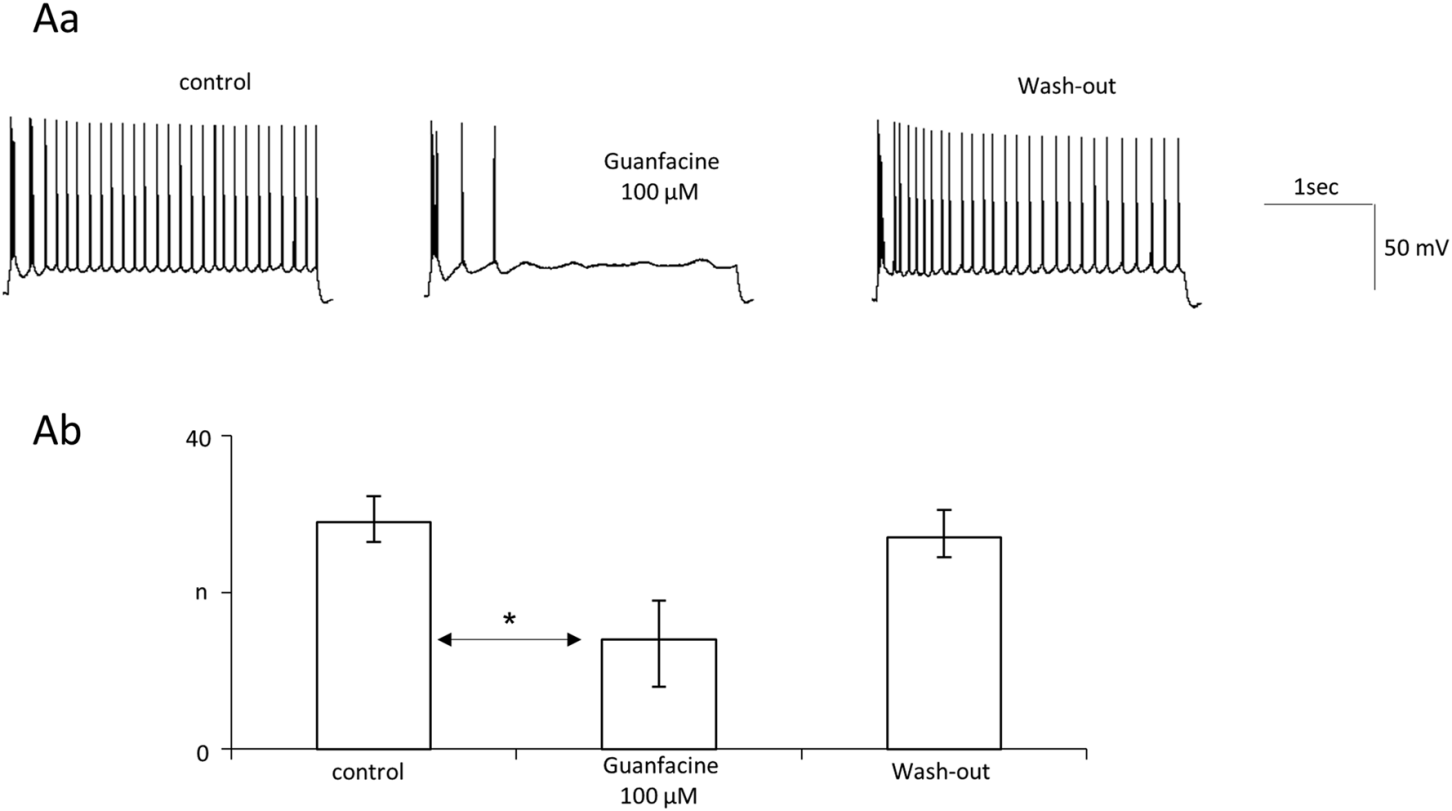
Guanfacine inhibits neuronal excitability in prefrontal cortex pyramidal neurons. **Aa** Example recordings of action potentials in the control, in the presence of guanfacine 100 µM and after wash-out. **Ab** Excitability (number of action potentials per current step) in the control, after application of the tested drug and after wash-out (Friedman’s test [control, drug application, wash-out], *p* = 0.0046, followed by Dunn’s post hoc test [control vs drug application], *p* < 0.05). Bars represent medians and whiskers represent IQR. *p < 0.05

In the next series of experiments, we recorded action potentials in PFC pyramidal neurons in physiological ACSF. Excitability was defined as the number of action potentials per depolarisation step lasting 3 s. We found that guanfacine 100 μM inhibited neuronal excitability (29.0 [26.5–32.3] in the control, 14.0 [8.0–19.0] in the presence of the tested drug and 27.0 [24.5–30.5] after wash-out, 2 animals, *n* = 4 recordings, nonparametric repeated measures ANOVA (Friedman’s test), Friedman’s statistic = 8, *p* = 0.0046 followed by Dunn’s post hoc test (*p* < 0.05, control vs guanfacine)). Example recordings and averaged results are shown in Fig. 3Aa and b, respectively.

**Fig. 4:**
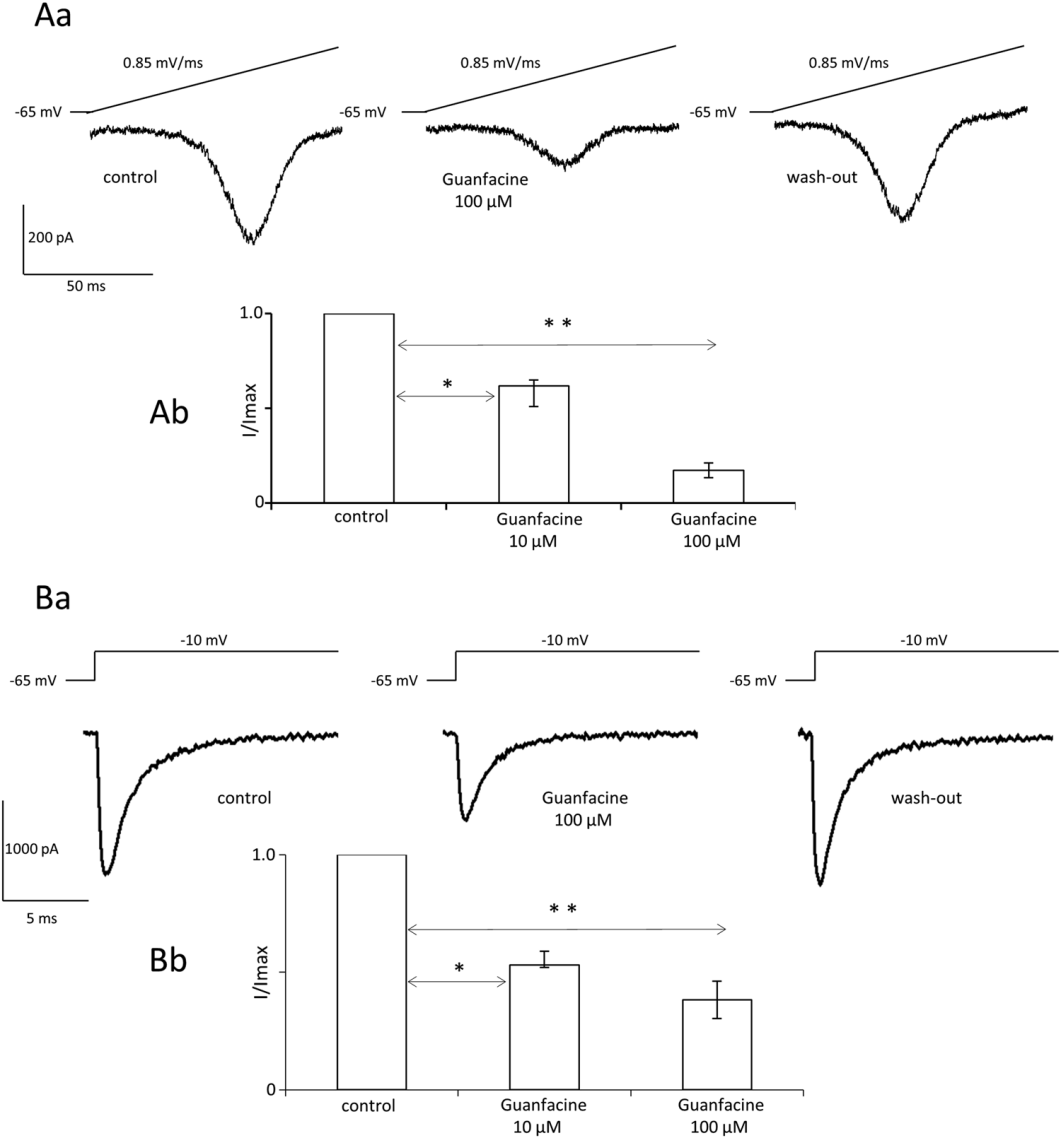
Guanfacine blocks persistent and fast inactivating sodium currents in prefrontal cortex pyramidal neurons. **Aa** Example recordings of slowly inactivating (persistent) sodium currents in the control, in the presence of guanfacine 100 µM and after wash-out. Persistent sodium currents were evoked by ramp depolarizations shown above current traces. **Ab** Normalized maximal persistent sodium current amplitudes in the control and after application of two concentrations of guanfacine: 10 µM (Friedman’s test [control, drug application, wash-out], *p* = 0.0046, followed by Dunn’s post hoc test [control vs drug application], *p* < 0.05, bars represent medians and whiskers represent IQR) and 100 µM (One-way ANOVA for repeated measures [control, drug application, wash-out], *p* < 0.0001, followed by Tukey’s post hoc test [control vs drug application], *p* < 0.001, bars represent means and whiskers represent SEM). *p < 0.05 and **p < 0.001. **Ba** Example recordings of fast inactivating (transient) sodium currents in the control, after application of guanfacine 100 µM and after wash-out. Sodium currents were evoked by rectangular voltage steps shown above current traces. **Bb** Normalized maximal transient sodium current amplitudes in the control and after application of two concentrations of guanfacine: 10 µM (Friedman’s test [control, drug application, wash-out], *p* = 0.0046, followed by Dunn’s post hoc test [control vs drug application], *p* < 0.05, bars represent medians and whiskers represent IQR) and 100 µM (One-way ANOVA for repeated measures, [control, drug application, wash-out] *p* < 0.0001, followed by Tukey’s post hoc test [control vs drug application], *p* < 0.001, bars represent means and whiskers represent SEM). *p < 0.05 and **p < 0.001

We hypothesized that guanfacine may inhibit persistent sodium currents because it has been found that the inhibition of these currents substantially contributes to decreasing excitability in central neurons [19]. Persistent voltage-gated sodium currents were evoked once every 20 s by short ramp depolarisations from − 65 mV to 10 mV lasting 100 ms. Control recordings were conducted for 2 min, guanfacine was applied for 3 min and after that wash-out was recorded. A dose-dependent effect was observed. A higher concentration of the tested drug (100 μM) inhibited persistent sodium currents (example recordings are shown in Fig. 4Aa). Normalized, maximal current amplitudes were 1.0 in the control, 0.17 ± 0.04 in the presence of guanfacine 100 μM and 0.66 ± 0.08 after wash-out (3 animals, *n* = 7 recordings, one-way ANOVA for repeated measures, *F*_3,20_ = 119.44 (*p* < 0.0001) followed by Tukey’s post hoc test (*p* < 0.001 control vs guanfacine)). Furthermore, normalized maximal current amplitudes were 1.0 [1.0–1.0], 0.62 [0.51–65] and 0.78 [0.55–0.82] in the control, after the application of guanfacine 10 μM and after wash-out, respectively (2 animals, *n* = 4 recordings, nonparametric repeated measures ANOVA (Friedman’s test), Friedman’s statistic = 8, *p* = 0.0046 followed by Dunn’s post hoc test (*p* < 0.05, control vs guanfacine)). Normalized results are depicted as medians in Fig. 4Ab.

In the next series of experiments, the influence of guanfacine on fast-activating and fast-inactivating voltage-gated sodium channels was tested. The currents were evoked once every 10 s by rectangular voltage steps to − 10 mV. Control recordings were conducted for 2 min, the tested drug was applied for 2 min and after that wash-out was recorded. Example recordings of fast inactivating sodium currents are shown in Fig. 4Ba. After the application of guanfacine 100 μM, the maximal, normalized sodium current amplitude was 0.38 ± 0.08 as compared to control 1.0 (Fig. 4Bb). It was possible to obtain wash-out (0.73 ± 0.08, 2 animals, *n* = 5 recordings, one-way ANOVA for repeated measures, *F*_3,14_ = 40.95 (*p* < 0.0001) followed by Tukey’s post hoc test (*p* < 0.001 control vs guanfacine)). A lower concentration of guanfacine (10 μM) was also tested and the maximal, normalized sodium current amplitudes were 1.0 [1.0–1.0] in the control, 0.53 [0.52–0.59] after application of the tested drug and 0.66 [0.64–0.71] after wash-out (2 animals, *n* = 4 recordings, nonparametric repeated measures ANOVA (Friedman’s test), Friedman’s statistic = 8, *p* = 0.0046 followed by Dunn’s post hoc test (*p* < 0.05, control vs guanfacine), Fig. 4Bb). We also assessed the time-dependent inactivation of fast voltage-gated sodium currents. Tau constants of time-dependent inactivation were not significantly different in control and in the presence of guanfacine 100 µM (1.6 ± 0.12 ms and 1.74 ± 0.22 ms, respectively, *n* = 4 recordings, paired t test *p* > 0.05, *t*_3_ = 0.6).

### Guanfacine does not influence tonic NMDA currents in PFC pyramidal neurons

**Fig. 5:**
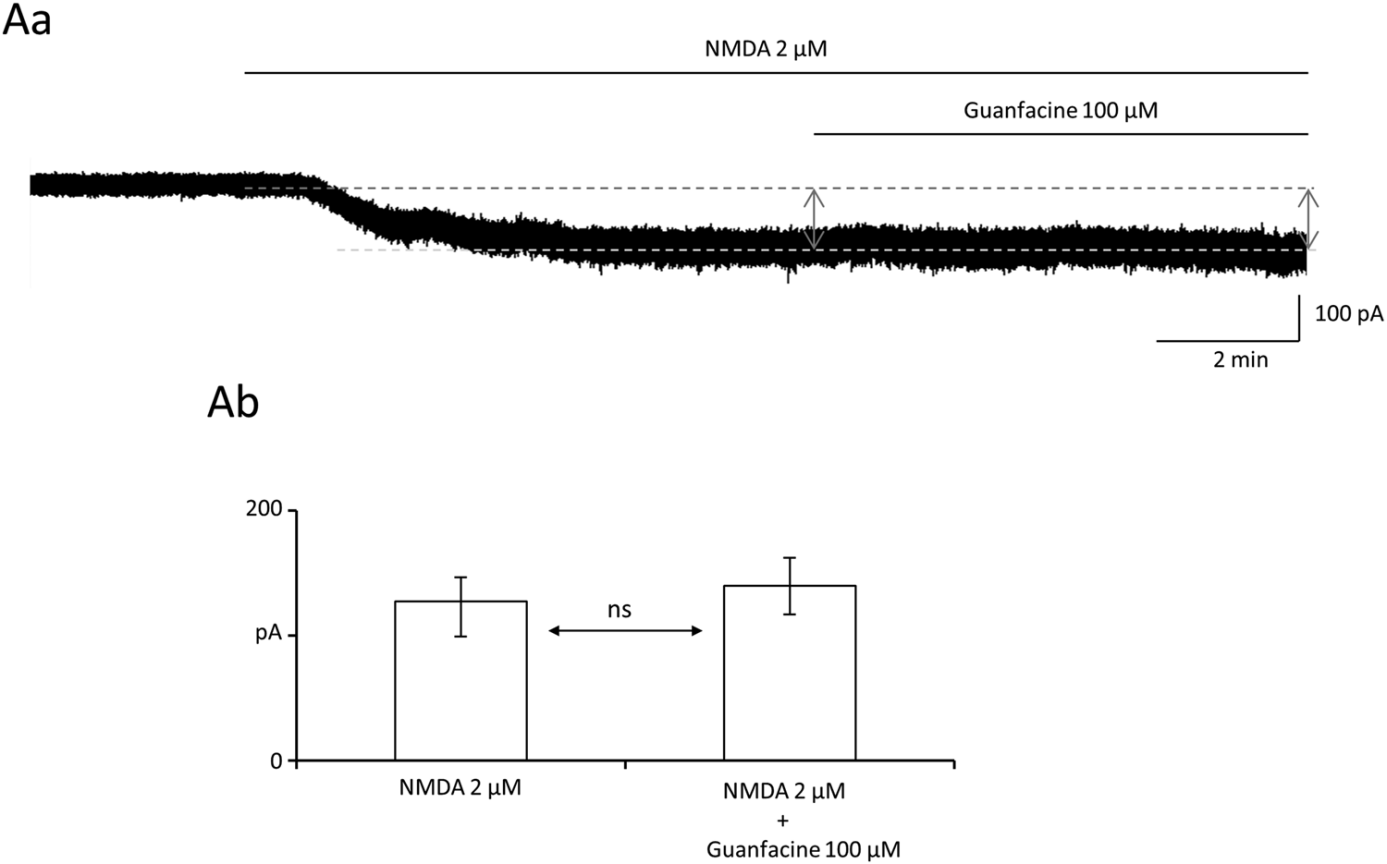
Guanfacine does not influence tonic NMDA currents. **Aa** Example recording of NMDA current evoked by application of NMDA 2 µM to the whole bath. Dashed lines indicate control current and current after application of NMDA 2 µM. Left vertical arrow indicates the NMDA current before the application of guanfacine and the right vertical arrow indicates the NMDA current after the application of guanfacine 100 µM. **Ab** NMDA current without guanfacine and NMDA current after application of guanfacine 100 µM (Wilcoxon’s matched-pairs test [control vs drug application], *p* > 0.05). Bars represent medians and whiskers represent IQR. *ns* nonsignificant

Recordings were conducted in an extracellular solution that contained no magnesium ions, glycine 50 µM, TTX 0.25 µM, DNQX 10 µM and picrotoxin 50 µM (see Methods). NMDA 2 µM without guanfacine was applied for 8–10 min. After evoking stable NMDA currents, NMDA 2 µM and guanfacine 100 µM were coapplied for 7 min. The amplitude of the control NMDA currents was 127.0 [99.3–146.3] pA, as shown by the left grey arrow in Fig. 5Aa. The amplitude of the NMDA currents after the application of guanfacine 100 µM was 139.5 [117.0 -162.0] pA as shown by the right grey arrow in Fig. 5Aa, which was not significantly different from the control NMDA currents (Fig. 5Ab *n*= 4 recordings, 2 animals, Wilcoxon’s matched-pairs test, *p* > 0.05). It was shown in our previous study [20] that tonic NMDA currents were fully inhibited by a selective NMDA inhibitor, AP-5.

## Discussion

The epileptiform discharges recorded in this study may be regarded as interictal events because of their short duration (less than 2 s) [9]. They do not cause seizures but are often recorded in EEG between seizures in epilepsy patients [9]. They can also be present in non-epileptic patients with different neuropsychiatric disorders [7, 10, 13–15].

In this study, IEDs were recorded with the use of the patch-clamp technique in PFC pyramidal neurons in a zero magnesium, elevated potassium pro-epileptic solution. Such composition of the extracellular solution enhances the effects of glutamate on NMDA receptors/channels because magnesium ions are removed from the NMDA channels pore. Furthermore, more glutamate is released from presynaptic terminals in the presence of increased potassium concentration because presynaptic axons are depolarized. Thus, the zero magnesium, elevated potassium extracellular solution increases the glutaminergic transmission that generates IEDs [16, 21].

We found that guanfacine blocks IEDs. The tested drug, however, did not exert this effect via the inhibition of glutaminergic NMDA receptors/channels, because we showed that they were not influenced by guanfacine. We recorded both synaptic and extra-synaptic NMDA currents simultaneously since the recordings were made from the whole plasma membrane, and NMDA was applied to the whole bath [22].

Glutamate release is caused by the opening of presynaptic voltage-gated sodium and calcium channels [23, 24]. Consequently, guanfacine may inhibit IEDs by targeting presynaptic voltage-gated sodium and calcium channels, thus lowering increased glutamate release, which generates IEDs. Additionally, guanfacine may block IEDs by suppressing postsynaptic sodium and calcium channels, as they were also reported to be involved in the generation of IEDs [25, 26]. There are two types of voltage-gated sodium currents: fast inactivating (transient) and slowly inactivating (persistent) sodium currents [19]. In this study, we recorded both fast and persistent voltage-gated sodium channels from dispersed PFC pyramidal neurons and found that guanfacine inhibits these channels, which may substantially contribute to the blocking of IEDs by the tested drug.

Few reports assess guanfacine’s influence on the electrophysiological properties of neurons. It was found that guanfacine suppressed excitatory postsynaptic currents in PFC pyramidal neurons [18, 27]. Similarly, in vivo experiments showed that the application of guanfacine reduced field excitatory post-synaptic potentials in PFC neurons [18]. The reports cited above suggest that guanfacine inhibits glutaminergic transmission in PFC neurons via the α2A adrenergic receptors [18, 27]. The authors hypothesized that this mechanism may improve PFC functioning (working memory) during excessive stress. Different authors performed in-vivo experiments and found that guanfacine improved working memory by enhancing neuronal activity in the PFC during the delay period of a working memory task [28]. This effect was also abolished by the alpha-2 adrenergic receptor antagonist. The authors suggested that this mechanism may explain guanfacine’s beneficial effects in treating ADHD [28].

As stated above guanfacine enhances neuronal activity in the PFC during the delay period of a working memory task via alpha-2 adrenergic receptors [28]. It may be argued that mentioned result contradicts our study that shows that guanfacine inhibits neuronal excitability and IEDs via direct inhibition of sodium channels. It may, however, be hypothesized that a lower concentration of guanfacine may enhance neuronal activity via alpha-2 adrenergic receptors as shown previously [28] and a higher concentration of the tested drug may inhibit sodium channels and consequently block IEDs and neuronal excitability, as shown in the present study. The concentrations of guanfacine that we used were 10 µM and 100 µM and were higher than the therapeutic plasma concentration of guanfacine [29]. They were, however, similar to previous patch-clamp studies [18, 30, 31].

The most commonly described mechanism of action of guanfacine is stimulating G-protein-coupled alpha-2A adrenergic receptors [18, 27, 28]. Guanfacine, however, may also have other, alpha-2 adrenergic receptor-independent mechanisms of action. In other words, guanfacine may influence ionic channels either directly or via G-protein-coupled alpha-2 adrenergic receptors. We hypothesize that in our experiments, the effects of guanfacine were mediated via direct action on ionic channels due to the following reasons. Firstly, our experiments in slices showed that the tested drug inhibited IEDs in the presence of the selective alpha-2 adrenergic receptor antagonist. Secondly, in our experiments in dispersed neurons, guanfacine most likely directly inhibited sodium channels since fluoride ions in the patch pipette disrupted G-protein-mediated signalling [32]. The important finding of this study is that guanfacine may act not only by stimulating alpha-2A adrenergic receptors but also by an additional mechanism, which is the direct inhibition of sodium channels. Interestingly, the chemical structure of guanfacine, with an aromatic ring linked to an amine group by an amide bond, resembles local anaesthetics (sodium channel inhibitors). This strengthens our hypothesis that guanfacine directly influences sodium channels. There are reports showing that other alpha-2 adrenergic receptor agonists such as clonidine and dexmedetomidine block sodium channels in peripheral neurons and in cell lines in an adrenergic receptor-independent fashion [33–35].

IEDs occur more often in patients with ADHD and may contribute to symptoms of this disease [10–15]. There are clinical studies suggesting that antiepileptic drugs (sodium and calcium channel inhibitors) reduce ADHD symptoms. For example, it was found that the calcium channel inhibitor levetiracetam inhibits IEDs and reduces symptoms of ADHD in children suffering from this disease [11, 12]. Another study showed that sodium channel inhibitor lamotrigine decreases ADHD symptoms in epileptic patients with ADHD. This effect correlated with EEG normalization and a reduction of epilepsy symptoms [36]. It was also found that sodium channel inhibitor carbamazepine inhibits IEDs in children with ADHD. This effect correlated with clinical improvement [37]. It could be speculated that in some patients guanfacine may reduce ADHD symptoms by inhibiting interictal epileptic events. Thus, guanfacine may exert beneficial effects in ADHD not only by stimulating alpha-2 adrenergic receptors as shown previously [6] but also in an additional mechanism which is the inhibition of sodium channels and consequently inhibition of IEDs.

This study shows that guanfacine inhibits IEDs in prefrontal cortex pyramidal neurons independently of alpha-2A adrenergic receptors. Sodium channel blockade by guanfacine is likely involved in this effect. This novel mechanism may be important clinically as inhibition of IEDs by guanfacine may reduce symptoms of ADHD.

## Supplementary Information

Below is the link to the electronic supplementary material.

Supplementary file1 (XLSX 14 KB)

## Data Availability

Raw data are provided in a supplementary file.
